# HER-2-Positive Tumors: A Continuously Evolving Field in Cancer Research

**DOI:** 10.3390/cancers15133333

**Published:** 2023-06-25

**Authors:** Ralf Hofheinz, Sylvie Lorenzen, Michael K. Bohlmann

**Affiliations:** 1 Mannheim Cancer Center, University Hospital Mannheim, University of Heidelberg, 68167 Mannheim, Germany; 2 Klinik und Poliklinik für Innere Medizin III, Technische Universität München, 81675 München, Germany; sylvie.lorenzen@mri.tum.de; 3 Zentrum für Geburtshilfe und Gynäkologie, St. Elisabethen Krankenhaus Lörrach, 79539 Lörrach, Germany; m.bohlmann@elikh.de

Almost 25 years ago, trastuzumab, a monoclonal antibody targeting the human epidermal growth factor receptor 2 (HER2), was licensed for the treatment of patients with metastatic HER2-positive breast cancer in the United States of America (USA). Since then, HER2-targeted treatment for patients with HER2-positive breast cancer has been substantially improved in several regards: (i) HER2-targeted therapy has moved from the palliative into the adjuvant and later on into the neoadjuvant treatment setting; (ii) double targeting using pertuzumab in combination with trastuzumab has been implemented into the metastatic as well as into the neoadjuvant treatment setting; (iii) tyrosine kinase inhibitors such as lapatinib, tucatinib, and neratinib have been introduced into the treatment of patients suffering from metastatic disease, partially in combination with trastuzumab, enabling HER2-directed treatment in several lines of treatment; (iv) antibody–drug conjugates have been approved for the treatment of breast cancer, namely trastuzumab emtansine and trastuzumab deruxtecan; and (v) the latter drug has also been shown to exhibit substantial efficacy in patients with tumors not fulfilling the “classical” criteria for HER2 positivity (i.e., HER2 3+ and HER2 2+ with ISH positivity). Patients with so-called HER2-low tumors (i.e., HER2 1+ or HER2 2+, ISH negativity) were shown to have a significant survival advantage over former standard treatments when trastuzumab deruxtecan was given as a second-line treatment. As a consequence, this drug is meanwhile approved for the treatment of these patients.

In all, the evolution of HER2-positive breast cancer is a success story and served as a blueprint for the development of HER2-targeted medical treatment also in other HER2-positive tumors. Firstly, in metastatic gastric cancer adding trastuzumab to platinum/5-fluorouracil-based treatment led to a significant survival benefit in the pivotal ToGA trial in 2010 [1]. Yet, the double targeting with pertuzumab and trastuzumab did not statistically increase survival in comparison with the ToGA regimen in the JACOB study [2]. Moreover, other randomized trials using lapatinib [3] and trastuzumab emtasine [4] delivered negative results, as well. Trastuzumab deruxtecan, however, is meanwhile licensed as a second-line treatment based on phase II trials [5].

Determination of HER2 expression in gastric cancer, however, represents still a challenge as gastric cancer tumor cells have greater HER-2 heterogeneity compared to breast cancer. Recently, the VARIANZ study demonstrated that only patients with centrally confirmed HER2 positivity appeared to derive benefit from a trastuzumab-based treatment due to a higher rate of tumors testing as false positives locally [6]. In terms of pathological workup of tumor tissue, it is known that different criteria for HER2 positivity are in place for gastric as well as breast cancer. For colorectal cancer—the third entity in which HER2 targeted treatment was investigated—a different scoring was proposed by Valtorta and coworkers [7]. In patients with HER2-positive metastatic colorectal cancer (mCRC), several phase II trials demonstrated promising progression-free and overall survival rates, mostly in heavily pretreated patients. For instance, in a recent phase II trial, a combination of tucatinib and trastuzumab yielded a response rate of 38% and led to a PFS of 8.2 months in 84 patients with mCRC with at least two lines of pretreatment [8]. This combination has meanwhile been licensed in the USA for the treatment of mCRC. Metastatic biliary tract cancer (BTC), especially gallbladder and extrahepatic BTC, is found to be HER2-positive in a substantial amount of patients. Recently, at the ASCO 2023 meeting, two phase II studies reported the activity of HER2-targeted therapies in this formerly difficult-to-treat population [9,10]. For instance, a phase II trial using zanidatamab, a bispecific monoclonal antibody targeting two domains of the HER2 receptor, reported a response rate of 41%, a median of PFS 5.5 months, and a duration of remission of about 1 year [9].

The current Special Issue of *Cancers* comprises five publications broadening our understanding of the diagnosis and treatment of HER2-positive cancers. The mode of action and results of HER2-directed drugs including recently approved drugs were reviewed in depth by Morales and coworkers [11]. New combinations of drugs targeting HER2 on one and other targets on the other hand are being explored in different tumor types in an attempt to increase efficacy and/or to prevent escape mechanisms. Using an ovarian cancer xenograft model expressing EpCAM and HER2, Xu and coworkers sought to assess the efficacy of a double targeting strategy. They demonstrated that the combination was able to optimize tumor shrinkage and improve survival in comparison to the monotherapy or no treatment [12].

The combination of HER2-targeted therapies with radiotherapy has been investigated in very few (randomized) trials. Disappointingly, in HER2-positive locally advanced adenocarcinomas of the gastro-esophageal junction, for instance, no benefit was found while using trastuzumab in combination with standard radiotherapy [13]. Debbi and coworkers discussed the background and safety of this combination and potential pitfalls in a comprehensive review of patients with breast cancer. They concluded that monoclonal antibodies and checkpoint inhibitors can safely be combined with radiation, while they raised caution in terms of the combination of tyrosine kinase inhibitors and antibody drug conjugates with radiation [14].

Two papers in this Special Issue deal with the neoadjuvant treatment of breast cancer. Tokunaga and colleagues reported the long-term results of the five-arm randomized phase II Neo-Lath Study [15]. This trial sought to assess the benefit of prolonged neoadjuvant HER2 targeted treatment and reports on the 5-year results. Harbeck and coworkers performed a biomarker study from patients with HER2-positive early breast cancer included in the neoadjuvant WSG-ADAPT HER2 study. This analysis identified patients with treatment-induced CD8 protein-expressing cells without PIK3CA mutation who may qualify for less intensive treatment after neoadjuvant therapy and tumor resections [16].

In summary, 25 years after the approval of trastuzumab for the treatment of metastatic breast cancer, we face a broad and sequential use of different HER2-targeted drugs in gastric cancer and breast cancer patients, for the latter even in HER2-low tumors. The use of HER2-targeted treatment in other gastrointestinal tumors was shown to give favorable results in phase II trials and will broaden the treatment spectrum for patients suffering from these tumors.

Although HER2-targeted therapy has dramatically improved the prognosis for patients with early- and late-stage HER2-positive tumors, acquired resistance to trastuzumab due to ERr2 loss as well as increased heterogeneity of HER2 gene expression are major obstacles in the treatment algorithm of HER2-positive tumors. Novel HER2-targeted drugs such as antibody–drug conjugates, tyrosine kinase inhibitors, and bispecific antibodies were developed to overcome these challenges. Besides this, new screening methods, such as ctDNA and new imaging agents (^89^Zr-Trastuzumab PET/CT), may allow real-time assessment and monitoring of anti-HER2 treatment in a less invasive manner in the future.
